# Hydration-Dehydration Effects on Germination Tolerance to Water Stress of Eight *Cistus* Species

**DOI:** 10.3390/plants14142237

**Published:** 2025-07-19

**Authors:** Belén Luna

**Affiliations:** Facultad de Ciencias Ambientales y Bioquímica, Universidad de Castilla-La Mancha, Av. Carlos III s/n, 45071 Toledo, Spain; belen.luna@uclm.es

**Keywords:** Cistaceae, drought resistance, fire, heat shock, hydration memory, Mediterranean, physical seed dormancy, priming

## Abstract

Seeds in soil are often exposed to cycles of hydration and dehydration, which can prime them by triggering physiological activation without leading to germination. While this phenomenon has been scarcely studied in wild species, it may play a critical role in enhancing drought resilience and maintaining seed viability under the warmer conditions predicted by climate change. In this study, I investigated the effects of hydration–dehydration cycles on germination response under water stress in eight *Cistus* species typical of Mediterranean shrublands. First, seeds were exposed to a heat shock to break physical dormancy, simulating fire conditions. Subsequently, they underwent one of two hydration–dehydration treatments (24 or 48 h) and were germinated under a range of water potentials (0, –0.2, –0.4, –0.6, and –0.8 MPa). Six out of eight species showed enhanced germination responses following hydration–dehydration treatments, including higher final germination percentages, earlier germination onset (T_0_), or increased tolerance to water stress. These findings highlight the role of water availability as a key factor regulating germination in *Cistus* species and evidence a hydration memory mechanism that may contribute in different ways to post-fire regeneration in Mediterranean ecosystems.

## 1. Introduction

Very different types of organisms, such as animals, bacteria, fungi, and plants, experience environmental stresses, which can help them cope with future stressful situations [1]. These organisms have the ability to remember past experiences and may prepare or “prime” the response against future environmental stimuli [2]. Overall, the term “priming” is used to describe this process by which organisms exposed to stress prepare and develop resistance for improving their responses against future stress. As environmental conditions change with global warming [3], this can impose serious problems for seed germination and, consequently, for the maintenance of plant communities and species persistence [4,5,6]. To assess the species’ vulnerability to climate change, it is essential to evaluate their plasticity to face the new environmental conditions and their mechanisms for enhancing stress responses.

Mediterranean areas are characterized by seasonal climates with periodic droughts and highly variable precipitation patterns, which are expected to intensify under future climate projections [3,7]. In these habitats, many plant species regenerate exclusively through seed germination, making the time of germination critical for the successful establishment of plants, and it is precisely regulated by environmental factors [8]. In the Mediterranean region, germination occurs mainly in autumn after the first rainfall once the soil is wet [9,10,11]. This timing is primarily regulated by water availability [12,13], with species showing specific water potential requirements to initiate germination [8,14]. Seeds in the soil start to hydrate when a rain event occurs and, when sufficient moisture is present, seeds may germinate [15]. However, if seeds are dormant or environmental conditions are not appropriate for germination, the seeds will dry as the soil dries. Furthermore, when water is limited water uptake may start, but the germination process cannot be completed. Thus, buried seeds in soil are subjected to cycles of hydration–dehydration before germination finally occurs, often under water stress conditions.

The germination process starts with seed imbibition, occurring in three distinct phases [16]. The first phase consists of a very fast initial uptake of water related to the passive imbibition of dry tissues. This is followed by a plateau phase, during which metabolism processes are re-established. Finally, the third phase marks an increase in water uptake, related to cell elongation and ultimately, radicle protrusion. If any of these three imbibition phases are not completed, germination cannot be concluded.

In germination ecophysiology, seed priming is a water-based technique involving controlled seed hydration and subsequent dehydration. Its purpose is to trigger the metabolic processes typically activated during the early phase of germination (“pre-germinative metabolism”), without allowing the seed to fully germinate or undergo radicle emergence [17]. Critically, this treatment must be stopped before the loss of desiccation tolerance occurs. This process effectively mimics the natural hydration–dehydration cycles experienced by seeds in the soil [18].

The mechanisms by which seeds improve their responses to stress have been extensively studied but not fully understood [19,20,21]. Germination increase after priming is related to improved metabolic and antioxidant activity within the seeds, which favors reparative processes [22,23]. Overall, seed priming or hydration–dehydration cycles can improve seed germination and stress tolerance through two main strategies [24,25]. First, seed priming promotes pre-germination metabolic processes. After wetting and drying, seeds are in a more advanced physiological state, facilitating the rapid restart of metabolic processes during subsequent imbibition. Second, seed priming may generate moderate abiotic stress during both hydration and dehydration periods, which allows the seeds to cope with environmental stresses. These two strategies constitute a “priming memory”, which can be employed after subsequent stress exposure, enhancing the stress tolerance when germinating primed seeds.

The effects of priming have been widely studied in crop species since efficient germination involves important economic and agronomic advantages [26,27]. However, its application and study in wild species has been scarce [28,29]. Seed priming leads to enhanced, rapid, and uniform germination [30], and has even more beneficial effects under unfavorable conditions. Seed priming has proved to be an effective method in improving stress tolerance against adverse conditions such as low water availability [31,32], salinity [33,34], or extremely high and low temperatures [35,36,37].

This investigation studies the effects of two different cycles of hydration–dehydration on the seed germination of eight *Cistus* species. *Cistus* species are widely represented in the Mediterranean shrublands, occupying vast extensions in open dry sunny habitats, especially after fire occurrences [38]. *Cistus* species have seeds with hard coats that impose physical dormancy (PY) [39]. PY break is made possible by seed coat scarification, and in fire-prone habitats is related to the heat produced during fires [39,40,41]. Once PY is broken, germination occurs under a wide range of environmental conditions [39,42,43], but different factors such as temperature, light, and water appear to regulate seed germination after PY release [12,44]. In this work, the effects of hydration–dehydration on seed germination are analyzed in relation to the germination response to water stress. In these environments, where in summer a water deficit limits growth and survival, an early establishment of seedlings may allow for greater root development to occur over the summer [45]. Moreover, being the first to occupy the space after the passage of fire may provide a competitive advantage in accessing resources. It could be expected that these species possess hydration memory, which enables rapid colonization following fire events. In other words, the exposure of seeds to precipitation events prior to germination may confer an advantage for subsequent germination, potentially allowing them to germinate under conditions of reduced water availability.

## 2. Results

**Figure 1:**
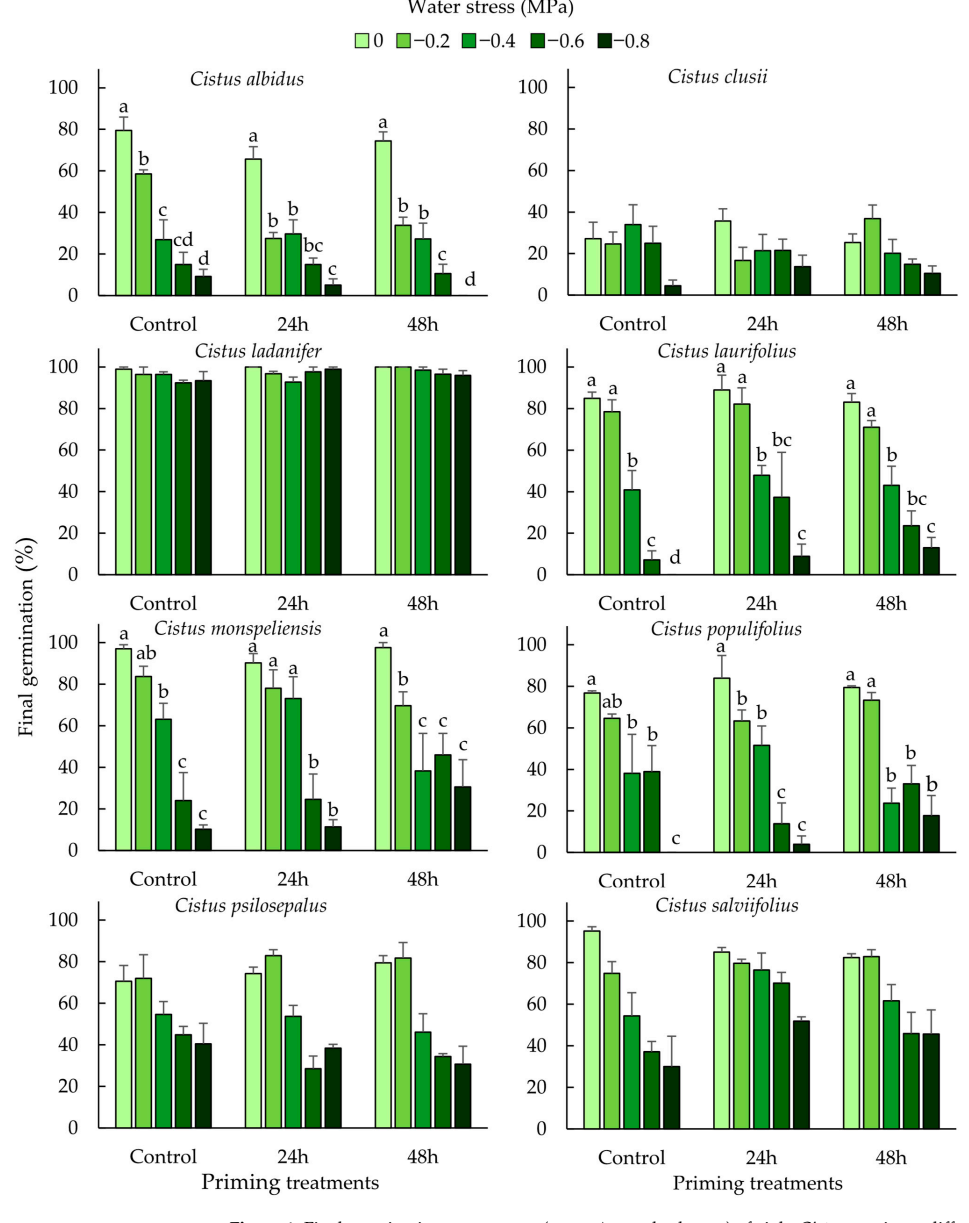
Final germination percentages (mean ± standard error) of eight *Cistus* species at different priming (control and hydration–dehydration cycles of 24 and 48 h) and water stress treatments (0, −0.2, −0.4, −0.6, and −0.8 MPa). Different letters show significant differences (*p* < 0.05) between water stress treatments based on pairwise comparisons of Fisher’s least significance difference after GLM analysis, when significant interactions between both factors emerged.

Water stress affected negatively the final germination of all *Cistus* species (Table 1; Figure 1) and delayed the onset of the germination of *C. albidus*, *C. ladanifer*, and *C. populifolius* (Table 2 and Table 3). That is, the final germination decreased with an increase in PEG water potentials for both primed and non-primed seeds. *C. ladanifer* showed the highest final germination, with a slight reduction even at the lowest water potential, while *C. clusii* showed very low germination percentages in all the treatments (Figure 1).

Priming had significant effects on the seed germination of five species (Table 1 and Table 3). These effects were negative only in the case of *C. albidus*, which had a decreased final germination value. In the cases of *C. ladanifer*, *C. laurifolius*, and *C. populifolius*, the final germination value increased and T_0_ decreased (Figure 1 and Figure S1, Table 2). Although the final germination of *C. psilosepalus* was not affected by priming, its T_0_ value also decreased. Furthermore, priming modified the response to water stress in six species; that is, there was a statistically significant interaction between priming and water stress (Table 1 and Table 3). Priming led to improved final germination under water stress in *C. laurifolius*, *C. monspeliensis*, and *C. populifolius*, a trend opposite to that observed in *C. albidus* (Figure 1). In the cases of *C. laurifolius* and *C. populifolius*, priming treatments widened the germination to −0.8 MPa, which was nil in the control treatment (Figure 1). In the case of *C. monspeliensis*, germination at the lowest water potentials (−0.6 and −0.8 MPa) increased at the longest priming treatment time (48 h). Additionally, priming induced early germination under water stress in *C. ladanifer*, *C. populifolius*, and *C. salviifolius* (Table 2 and Table 3).

## 3. Discussion

Water stress reduced germination both in non-primed and primed seeds, though the intensity of this reduction varied among species. Furthermore, water stress delayed the seed germination for three of the studied species. These findings align with the fundamental understanding that while sufficient moisture is essential for germination, each species has a critical water potential requirement for germination [8,14].

*C. ladanifer* exhibited the highest germination percentage across all the water potential levels. This low response to water stress contrasts with previous work, where gemination was reduced to 60% at −0.8 MPa [12]. On the contrary, the germination of non-primed seeds of *C. laurifolius* and *C. populifolius* was completely inhibited at the lowest water potential (−0.8 MPa). This differential sensitivity could be related to the higher precipitation levels at the sites of seed provenance of these two species, which exceed 1000 mm, compared to less than 500 mm in the case of *C. ladanifer* (Table 4). This supports the general idea that seeds from dry environments germinate at lower potentials than those from wet environments [46,47]. However, it is important to note that this relation is not always straightforward. In this sense, Chamorro et al. [13] found that although the seeds of *Erica arborea* from drier sites were able to germinate under lower water potentials compared to those from less arid sites, no such relationship was observed for *Cistus monspeliensis* and *C. salviifolius*, whose germination sensitivity to water stress did not correlate with the local climatic conditions of their provenance. Thus, further studies are needed to elucidate how maternal effects influence germination sensitivity to water stress within the Cistaceae species.

While the final germination value decreased with water stress for both primed and non-primed seeds, priming significantly alleviated this effect, i.e., the reduction in germination was more pronounced in non-primed seeds. Priming not only improved germination, but it modified the response to water stress in five species, expanding the germination requirements to a wider range of environmental conditions and promoting germination in suboptimal drought conditions [48,49]. The change in response to water stress was different among the different species. Priming widened the germination response of *C. laurifolius* and *C. populifolius* to −0.8 MPa, while for *C. monspeliensis*, germination under the lowest water potentials (−0.6 and −0.8 MPa) increased following the longest priming treatment time (48 h).

Surprisingly, an unexpected negative effect of seed priming was observed for *C. albidus*. The duration and frequency of hydration periods play a key role in determining whether their effects on germination are beneficial or detrimental [14]. Perhaps shorter or additional cycles of hydration–dehydration could promote the germination of *C. albidus*. While many species exhibit enhanced germination following cycles of hydration and dehydration, in other species, periods of dehydration may reduce germination or induce dormancy when compared to constant hydration [50,51], especially after prolonged periods of dehydration [52,53]. A threshold exists in the germination process, beyond which dehydration exerts detrimental effects on seeds [54]. Seed viability loss occurs when seeds absorb enough water to start cell division but not enough to finish the germination process [14]. In this sense, Wilson and Witowski [55] reported viability loss in *Acacia tortilis* and *A. nilotica* after specific hydration–dehydration cycles [55]. However, in the present research, seed viability loss was not the reason for the reduction in *C. albidus* germination with priming (Tables S1 and S2). On the other hand, water stress reduced seed viability in six out of the eight species, with four showing statistically significant differences and two exhibiting marginally significant differences. This loss of seed viability agrees with previous results for Cistaceae [12].

This study highlights the importance of water as a limiting factor for the germination of *Cistus* species in the Mediterranean. Water stress can be seen as a double-edged sword. While it has a well-documented deleterious effect on seed germination, it can also act as a priming factor, enhancing the plant’s ability to withstand future drought conditions compared to those that have not previously experienced such stress. In Mediterranean areas, where precipitation is highly variable and droughts are frequent, water availability controls plant regeneration [56,57]. *Cistus* is a very common genus of Mediterranean shrublands, which thrives especially after fire conditions because the physical dormancy of its seeds is broken with the high temperatures [39,58]. Fires in the Mediterranean region typically occur during the summer season [59,60], when temperatures are high and precipitation is limited, occurring only as sporadic stormy rains. Under such unfavorable conditions, germination is not possible and it is delayed until environmental conditions become suitable for germination and seedling establishment, i.e., in autumn [9,10]. In Mediterranean shrublands, germination is highest during wet years, occurring primarily in the first post-fire year. In contrast, during dry years, germination is reduced and can extend over several years [61]. Germination sensitivity to water stress can regulate the timing of germination and consequently improve reproductive success. The timing of germination influences the entire lifespan of the adult plant with consequences for survival, growth, and fecundity [62]. Fires open a narrow window that is free of competitors for resources where early germination is favored by natural selection [63]. Under these conditions, mechanisms that optimize early germination, like hydration memory, are favored.

*Cistus* seeds are small and must be kept close to the soil surface to germinate and become established [64]. It is in the soil surface or in the very upper soil layers where seeds are subjected to more pronounced water fluctuations, with more rapid wetting after rainfall, but also faster drying [65,66]. So, after fire occurrence, if sporadic rain events occur, *Cistus* seeds in the soil may hydrate partially and dehydrate until a new rain event [67]. Brief rain showers will ensure seeds remain partially hydrated without germinating, a strategy considered to be a type of dormancy and one that appears well-suited for dry habitats [68]. The effects of seed hydration–dehydration cycles are cumulative and would permit the acceleration of germination once adverse conditions disappear for plant establishment. In these environments, any strategy that reduces the time lag between the beginning of seed imbibition and seed germination is likely to be critical for successful seedling recruitment, ensuring fast colonization and appropriate root development for facing summer drought [45]. Hydration memory seems to be more common in arid environments, but it is also present in more mesic ones [28]. Most of the *Cistus* seeds examined in this study, which come from locations with different precipitations, exhibited hydration memory, a strategy that can facilitate post-fire regeneration when rainfall is enough and ultimately the conditions are appropriate for seedling establishment. Considering that physical seed dormancy is a recognized adaptation for plant survival in harsh and stochastic environments [69], the presence of these traits in *Cistus* might imply a certain germination resilience to the drier and more variable climates predicted with climate change. However, the ultimate impact on seedling survival and plant establishment, crucial for long-term population persistence, remains unknown.

## 4. Material and Methods

### 4.1. Study Species and Germination Experiments

The species studied were *Cistus albidus* L., *C. clusii* Dunal, *C. ladanifer* L., *C. laurifolius* L., *C. monspeliensis* L., *C. populifolius* L., *C. psilosepalus* Sweet, and *C. salviifolius* L. Fruits of all the species were harvested in the center of the Iberian Peninsula (Table 4), where the climate is Mediterranean. Although the potential vegetation is holm-oak woods in Spain (*Quercus rotundifolia* Lam.), the landscape is usually dominated by shrublands, which frequently show a high representation of Cistaceae species. Fruits were collected when ripened from at least 30 plants. Then, capsules were carried to the Ecology laboratory at the University of Castilla-La Mancha, where the seeds were separated and stored in paper bags at laboratory conditions. Cistaceae is a plant family characterized by hard seed coats, which impose the seed’s physical dormancy [39]. Therefore, prior to germination experiments, seeds were exposed to a heat shock of 100 °C for 10 min in an air-forced oven. These temperatures and times are commonly found in Mediterranean fire shrublands [10,61] and, this was a homogeneous optimal temperature for breaking seed dormancy of most of the studied species [70]. Prior to germination under different water stress conditions, seeds were hydrated–dehydrated for two periods of 24 and 48 h at the laboratory. Seeds were placed in plastic petri dishes 5.5 cm in diameter over two sheets of filter paper wetted with 2 mL of water. Petri dishes were sealed with parafilm to avoid desiccation. After 24 or 48 h, petri dishes were opened and the moistened filter paper was replaced by dry paper, thus allowing the seeds to dry for the respective periods of 24 or 48 h. Then, after these hydration–dehydration periods, or priming treatments, seeds were germinated in a temperature and humidity-controlled chamber (Model G-21, Ibercex) at 20 °C at a 12 h photoperiod (110.2 ± 4.2 µmol m^−2^ s^−1^), which is an optimum temperature for the germination of many Mediterranean species [42,71]. Relative humidity in the chamber was maintained at approximately 80%. Four replicates of 25 seeds were set to germinate for each species. Seeds were germinated under different levels of water stress by moistening the petri dishes with 1.2 mL of deionized water or the appropriate polyethylene glycol solution (PEG) to produce five levels of water potentials: 0, −0.2, −0.4, −0.6, and −0.8 MPa. The required water potential was produced with PEG 6000 and deionized water [72]. Germination was monitored daily for the first 15 days and every 3 days during the rest of the experiment, which lasted 42 days. At the end of the experiment, the viability of the ungerminated seeds was checked by a cut test. Seeds were cut with a scalpel and those seeds with a firm white endosperm were considered viable. Germination data were corrected by viability; that is, the final germination was assessed in relation to the viable seeds and not in relation to the total number of sown seeds.

### 4.2. Data Analyses

The final germination and time to onset of germination (T_0_) of each species were tested for the effects of priming and water stress treatments by means of generalized linear models (GLMs). Based on error structure, we used a binomial error distribution and logit link function for the final germination. In the case of T_0_, a Poisson error distribution with a log link function best fit the data. When significant differences were found, pairwise comparisons among treatments were performed using Fisher’s least significant difference post hoc test. All statistical analyses were performed using SPSS Statistics version 19.0 (SPSS, Chicago, IL, USA).

## Figures and Tables

**Table 1:** Results from the GLM for the main effects of water stress, priming factors, and their interactions on the final germination percentages of eight *Cistus* species. Statistically significant effects (*p* ≤ 0.05) are highlighted in bold.

	Water Stress			Priming			Water Stress x Priming	
	χ2	*p*		χ2	*p*		χ2	*p*
*Cistus albidus*	651.960	**<0.001**		5021.420	**<0.001**		3015.860	**<0.001**
*C. clusii*	13.880	**0.008**		0.233	0.89		10.754	0.216
*C. ladanifer*	12.279	**0.015**		6.744	**0.034**		10.209	0.251
*C. laurifolius*	623.606	**<0.001**		272.493	**<0.001**		1318.145	**<0.001**
*C. monspeliensis*	85.021	**<0.001**		1.027	0.598		16.276	**0.039**
*C. populifolius*	351.412	**<0.001**		241.520	**<0.001**		1308.337	**<0.001**
*C. psilosepalus*	90.735	**<0.001**		0.421	0.810		9.252	0.322
*C. salviifolius*	57.059	**<0.001**		3.709	0.157		9.886	0.273

**Table 2:** T_0_ (number of days to initiate germination) mean values (±standard error) for eight *Cistus* species at different priming (control and hydration–dehydration cycles of 24 and 48 h) and water stress treatments (0, −0.2, −0.4, −0.6, and −0.8 MPa).

		Control			24 h			48 h		
*Cistus albidus*	0	11.00	±	1.08	11.00	±	1.08	15.00	±	5.02
	−0.2	16.00	±	2.12	16.75	±	3.33	15.00	±	2.89
	−0.4	27.25	±	6.02	19.50	±	7.89	18.50	±	6.55
	−0.6	19.67	±	9.17	29.25	±	7.82	19.00	±	10.21
	−0.8	5.00	±	2.52	9.50	±	1.50			
*C. clusii*	0	12.75	±	1.38	9.50	±	1.66	14.50	±	5.19
	−0.2	14.00	±	5.37	21.67	±	5.67	8.25	±	2.10
	−0.4	12.50	±	2.53	16.00	±	4.74	12.00	±	1.15
	−0.6	4.67	±	1.20	15.25	±	8.32	8.00	±	2.00
	−0.8	8.50	±	1.50	6.00	±	2.52	9.33	±	1.33
*C. ladanifer*	0	7.00	±	0.00	2.00	±	0.00	2.25	±	0.25
	−0.2	7.00	±	0.00	3.75	±	0.25	2.00	±	0.00
	−0.4	5.75	±	1.25	5.50	±	0.87	2.50	±	0.50
	−0.6	7.00	±	0.00	4.75	±	1.31	5.25	±	1.03
	−0.8	4.75	±	1.44	5.50	±	0.87	4.00	±	1.22
*C. laurifolius*	0	9.75	±	1.70	3.75	±	2.10	5.50	±	3.52
	−0.2	13.00	±	8.43	4.00	±	1.73	7.25	±	1.93
	−0.4	6.67	±	2.03	6.33	±	1.20	11.00	±	4.64
	−0.6	17.00	±	10.69	1.00	±	0.00	2.25	±	0.25
	−0.8	3.67	±	2.19	2.50	±	0.50	4.50	±	2.18
*C. monspeliensis*	0	7.25	±	0.25	8.50	±	0.50	7.75	±	0.75
	−0.2	9.00	±	0.41	6.00	±	1.35	7.25	±	0.25
	−0.4	16.00	±	5.82	6.25	±	1.80	8.50	±	0.65
	−0.6	9.00	±	4.51	15.50	±	7.53	7.50	±	2.25
	−0.8	9.00	±	1.15	10.50	±	0.87	5.25	±	2.02
*C. populifolius*	0	22.00	±	7.02	19.00	±	5.20	10.50	±	3.75
	−0.2	26.00	±	5.16	16.75	±	3.20	10.00	±	0.00
	−0.4	18.50	±	2.06	22.50	±	3.71	34.00	±	4.08
	−0.6	38.00	±	0.00	30.00	±	8.00	17.25	±	6.92
	−0.8				10.00			21.00	±	9.00
*C. psilosepalus*	0	12.50	±	1.50	10.00	±	0.41	10.75	±	3.09
	−0.2	12.25	±	1.65	9.00	±	0.41	13.75	±	2.29
	−0.4	12.25	±	1.75	7.50	±	2.25	16.50	±	4.56
	−0.6	12.50	±	0.87	9.50	±	0.65	10.50	±	1.44
	−0.8	10.50	±	2.02	8.50	±	2.72	13.25	±	1.49
*C. salviifolius*	0	8.00	±	1.68	10.25	±	0.63	8.25	±	0.25
	−0.2	10.00	±	0.00	9.25	±	0.48	8.50	±	0.29
	−0.4	10.00	±	0.41	6.75	±	1.03	8.50	±	0.50
	−0.6	9.00	±	0.00	6.25	±	1.44	9.75	±	0.25
	−0.8	11.33	±	2.40	9.25	±	0.75	7.00	±	2.04

**Table 3:** Results from the GLM for the main effects of water stress, priming factors, and their interactions on T_0_ (number of days needed until the beginning of germination) of eight *Cistus* species. Statistically significant effects (*p* ≤ 0.05) are highlighted in bold, while marginally significant effects are in italics (0.05 < *p* < 0.1).

	Water Stress			Priming			Water Stress x Priming	
	χ2	*p*		χ2	*p*		χ2	*p*
*Cistus albidus*	11.495	**0.022**		0.455	0.796		3.625	0.822
*C. clusii*	6.178	0.186		1.506	0.471		9.640	0.291
*C. ladanifer*	14.280	**0.006**		37.579	**<0.001**		24.786	**0.002**
*C. laurifolius*	3.586	0.465		5.751	*0.056*		7.530	0.481
*C. monspeliensis*	2.883	0.578		2.683	0.261		11.430	0.179
*C. populifolius*	11.594	**0.021**		5.194	*0.074*		19.845	**0.006**
*C. psilosepalus*	0.429	0.980		8.828	**0.012**		4.991	0.759
*C. salviifolius*	1.891	0.756		4.517	0.105		15.843	**0.045**

**Table 4:** Geographic coordinates and characterization of locations where *Cistus* seeds were harvested.

Species	Latitude	Longitude	Altitude	Temperature	Precipitation
	(N)	(W)	(m)	(°C)	(mm)
*Cistus albidus*	39.82°	4.24°	508	14.6	451
*C. clusii*	40.18°	3.54°	639	14.4	437
*C. ladanifer*	39.88°	4.24°	550	14.6	451
*C. laurifolius*	40.72°	4.03°	1246	10.3	1021
*C. monspeliensis*	39.64°	4.39°	682	13.9	396
*C. populifolius*	40.22°	5.20°	1552	8	1370
*C. psilosepalus*	40.03°	5.05°	375	16.2	617
*C. salviifolius*	39.82°	4.24°	508	14.6	451

## Data Availability

Data are contained within the article or Supplementary Material.

## Supplementary Materials

The following supporting information can be downloaded at: https://www.mdpi.com/article/10.3390/plants14142237/s1. Table S1: Seed viability for different treatments and species; Table S2: Results of GLM for seed viability; Figure S1: Final germination for the different treatments and species.

## References

[1] Hilker M., Schwachtje J., Baier M., Balazadeh S., Bäurle I., Geiselhardt S., Hincha D.K., Kunze R., Mueller-Roeber B., Rillig M.C. (2015). Priming and memory of stress responses in organisms lacking a nervous system. Biol. Rev..

[2] Bruce T.J.A., Matthes M.C., Napier J.A., Pickett J.A. (2007). Stressful “memories” of plants: Evidence and possible mechanisms. Plant Sci..

[3] IPCC (2023). Climate change 2022: Impacts, adaptation and vulnerability. Working Group II. Contribution of Working Group II to the Sixth Assessment Report of the Intergovernmental Panel on Climate Change.

[4] Thuiller W., Lavorel S., Araújo M.B., Sykes M.T., Prentice I.C. (2005). Climate change threats to plant diversity in Europe. Proc. Natl. Acad. Sci. USA.

[5] Walck J.L., Hidayati S.N., Dixon K.W., Thompson K., Poschlod P. (2011). Climate change and plant regeneration from seed. Glob. Change Biol..

[6] Pacifici M., Foden W.B., Visconti P., Watson J.E., Butchart S.H., Kovacs K.M., Rondinini C. (2015). Assessing species vulnerability to climate change. Nat. Clim. Change.

[7] Ruffault J., Martin-StPaul N.K., Duffet C., Goge F., Mouillot F. (2014). Projecting future drought in Mediterranean forests: Bias correction of climate models matters!. Theor. Appl. Climatol..

[8] Baskin C.C., Baskin J.M. (2014). Seeds. Ecology, Biogeography and Evolution of Dormancy and Germination.

[9] Espigares T., Peco B. (1993). Mediterranean pasture dynamics: The role of germination. J. Veg. Sci..

[10] Céspedes B., Torres I., Luna B., Pérez B., Moreno J.M. (2012). Soil seed bank, fire season, and temporal patterns of germination in a seeder-dominated Mediterranean shrubland. Plant Ecol..

[11] Chamorro D., Luna B., Moreno J.M. (2017). Germination responses to current and future temperatures of four seeder shrubs across a latitudinal gradient in western Iberia. Am. J. Bot..

[12] Luna B., Chamorro D. (2016). Germination sensitivity to water stress of eight Cistaceae species from the Western Mediterranean. Seed Sci. Res..

[13] Chamorro D., Luna B., Ourcival J.-M., Kavgaci A., Sirca C., Mouillot F., Arianoutsou M., Moreno J.M. (2017). Germination sensitivity to water stress of four shrubby species across the Mediterranean Basin. Plant Biol..

[14] Bewley J.D., Bradford K., Hilhorst H., Nonogaki H. (2013). Seeds. Physiology of Development, Germination and Dormancy.

[15] Batlla D., Benech-Arnold R.L. (2006). The role of fluctuations in soil water content on the regulation of dormancy changes in buried seeds of *Polygonum aviculare* L.. Seed Sci. Res..

[16] Finch-Savage W.E., Leubner-Metzger G. (2006). Seed dormancy and the control of germination. New Phytol..

[17] Bewley J.D., Black M. (1994). Seed Physiology of Development and Germination.

[18] González-Zertuche L., Vázquez-Yanes C., Gamboa A., Sánchez-Coronado M.E., Aguilera P., Orozco-Segovia A. (2001). Natural priming of *Wigandia urens* seeds during burial: Effects on germination, growth and protein expression. Seed Sci. Res..

[19] Lutts S., Benincasa P., Wojtyla L., Kubala S., Pace R., Lechowska K., Quinet M., Garnczarska M. (2016). Seed priming: New comprehensive approaches for an old empirical technique. New Challenges in Seed Biology-Basic and Translational Research Driving Seed Technology.

[20] Srivastava A.K., Suresh Kumar J., Suprasanna P. (2021). Seed ‘primeomics’: Plants memorize their germination under stress. Biol. Rev..

[21] Pagano A., Macovei A., Balestrazzi A. (2023). Molecular dynamics of seed priming at the crossroads between basic and applied research. Plant Cell Rep..

[22] Bewley J.D., Black M. (1982). Physiology and biochemistry of seeds in relation to germination. Viability, Dormancy and Environmental Control.

[23] Long R.L., Kranner I., Panetta F.D., Birtic S., Adkins S.W., Steadman K.J. (2011). Wet-dry cycling extends seed persistence by re-instating antioxidant capacity. Plant Soil.

[24] Chen K., Arora R. (2013). Priming memory invokes seed stress-tolerance. Environ. Exp. Bot..

[25] Paparella S., Araujo S.S., Rossi G., Wijayasinghe M., Carbonera D., Balestrazzi A. (2015). Seed priming: State of the art and new perspectives. Plant Cell Rep..

[26] Jisha K.C., Vijayakumari K., Puthur J.T. (2013). Seed priming for abiotic stress tolerance: An overview. Acta Physiol. Plant..

[27] Haj Sghaier A., Tarnawa Á., Khaeim H., Kovács G.P., Gyuricza C., Kende Z. (2022). The effects of temperature and water on the seed termination and seedling development of rapeseed (*Brassica napus* L.). Plants.

[28] Contreras-Quiroz M., Pando-Moreno M., Jurado E., Flores J., Bauk K., Gurvich D.E. (2016). Is seed hydration memory dependent on climate? Testing this hypothesis with Mexican and Argentinian cacti species. J. Arid Environ..

[29] Copete M.A., Herranz J.M., Herranz R., Copete E., Ferrandis P. (2021). Effects of desiccation of seeds in nine species with morphophysiological dormancy on germination and embryo growth. J. Plant Ecol..

[30] McDonald M.B., Black M., Bewley J.D. (2000). Seed priming. Seed Technology and its Biological Basis.

[31] Frett J.J., Pill W.G. (1989). Germination characteristics of osmotically primed and stored *Impatiens* seeds. Sci. Hortic..

[32] Zhang F., Yu J., Johnston C.R., Wang Y., Zhu K., Lu F., Zou J. (2015). Seed priming with polyethylene glycol induces physiological changes in sorghum (*Sorghum bicolor* L. Moench) seedlings under suboptimal soil moisture environments. PLoS ONE.

[33] Wiebe H.J.A., Muhyaddin T. (1987). Improvement of emergence by osmotic seed treatments in soils of high salinity. Acta Hortic..

[34] Ibrahim E.A. (2016). Seed priming to alleviate salinity stress in germinating seeds. J. Plant Physiol..

[35] Valdes V.M., Bradford K.J., Mayberry K.S. (1985). Alleviation of thermodormancy in coated lettuce seeds by seed priming. HortScience.

[36] Bradford K.J. (1986). Manipulation of seed water relations via osmotic priming to improve germination under stress. HortScience.

[37] Pill W.G., Finch-Savage W.E. (1988). Effects of combining priming and plant growth regulator treatments on the synchronisation of carrot seed germination. Ann. App. Biol..

[38] Allen H., Woodward J.C. (2009). Vegetation and ecosystem dynamics. The Physical Geography of the Mediterranean.

[39] Thanos C.A., Georghiou K., Kadis C., Pantazi C. (1992). Cistaceae: A plant family with hard seeds. Israel J. Bot..

[40] Aronne G., Mazzoleni S. (1989). The effects of heat exposure on seeds of *Cistus incanus* L. and *Cistus monspeliensis* L.. G. Bot. Ital..

[41] Keeley J.E., Pausas J.G., Rundel P.W., Bradstock R. (2011). Fire as an evolutionary pressure shaping plant traits. Trends Plant Sci..

[42] Thanos C.A., Georghiou K. (1988). Ecophysiology of fire-stimulated seed germination in *Cistus incanus* ssp. *creticus* (L.) Heywood and *C. salvifolius* L.. Plant Cell Environ..

[43] Baskin J.M., Baskin C.C., Li X. (2000). Taxonomy, anatomy and evolution of physical dormancy in seeds. Plant Spec. Biol..

[44] Luna B., Piñas-Bonilla P., Zavala G., Pérez B. (2022). Effects of light and temperature on seed germination of eight *Cistus* species. Seed Sci. Res..

[45] De Luis M., Verdú M., Raventós J. (2008). Early to rise makes a plant healthy, wealthy, and wise. Ecology.

[46] Evans C.E., Etherington J.R. (1990). The effect of soil-water potential on seed-germination of some British plants. New Phytol..

[47] Bao G., Zhang P., Wei X., Zhang Y., Liu W. (2022). Comparison of the effect of temperature and water potential on the seed germination of five *Pedicularis kansuensis* populations from the Qinghai-Tibet plateau. Front. Plant Sci..

[48] Lewandrowski W., Erickson T.E., Dalziell E.L., Stevens J.C. (2018). Ecological niche and bet-hedging strategies for *Triodia* (R.Br.) seed germination. Ann. Bot..

[49] Kildisheva O.A., Erickson T.E., Madsen M.D., Dixon K.W., Merritt D.J. (2019). Seed germination and dormancy traits of forbs and shrubs important for restoration of North American dryland ecosystems. Plant Biol..

[50] Downs M.P., Cavers P.B. (2000). Effects of wetting and drying on seed germination and seedling emergence of bull thistle, *Cirsium vulgare* (Savi) Ten. Can. J. Bot.-Rev. Can. Bot..

[51] Ren J., Tao L. (2003). Effect of hydration-dehydration cycles on germination of seven *Calligonum* species. J. Arid Environ..

[52] Walck J.L., Baskin J.M., Baskin C.C. (1997). A comparative study of the seed germination biology of a narrow endemic and two geographically-widespread species of *Solidago* (Asteraceae) 4. Role of soil moisture in regulating germination. Seed Sci. Res..

[53] Kagaya M., Tani T., Kachi N. (2005). Effect of hydration and dehydration cycles on seed germination of *Aster kantoensis* (Compositae). Can. J. Bot.-Rev. Can. Bot..

[54] Berrie A.M.M., Drennan D.S.H. (1971). Effect of hydration-dehydration on seed germination. New Phytol..

[55] Wilson T.B., Witkowski E.T.F. (1998). Water requirements for germination and early seedling establishment in four African savanna woody plant species. J. Arid Environ..

[56] Hadas A., Khan A.A. (1982). Water movement to seeds in soils and seed water uptake. The Physiology and Biochemistry of Seed Development, Dormancy and Germination.

[57] Koller D., Hadas A., Lange O.L., Nobel P.S., Osmond C.B., Ziegler H. (1982). Water relations in the germination of seeds. Physiological Plant Ecology II: Water Relations and Carbon Assimilation.

[58] Luna B., Chamorro D., Pérez B. (2019). Effect of heat on seed germination and viability in species of Cistaceae. Plant Ecol. Div..

[59] Vázquez A., Moreno J.M. (1998). Patterns of lightning-, and people-caused fires in peninsular Spain. Int. J. Wildland Fire.

[60] Vecín-Arias D., Castedo-Dorado F., Ordóñez C., Rodríguez-Pérez J.R. (2016). Biophysical and lightning characteristics drive lightning-induced fire occurrence in the central plateau of the Iberian Peninsula. Agric. For. Meteorol..

[61] Moreno J.M., Zuazua E., Pérez B., Luna B., Velasco A., de Dios V.R. (2011). Rainfall patterns after fire differentially affect the recruitment of three Mediterranean shrubs. Biogeosciences.

[62] Donohue K., de Casas R.R., Burghardt L., Kovach K., Willis C.G. (2010). Germination, postgermination adaptation, and species ecological ranges. Annu. Rev. Ecol. Evol. Syst..

[63] Verdú M., Traveset A. (2005). Early emergence enhances plant fitness: A phylogenetically controlled meta-analysis. Ecology.

[64] Traba J., Azcárate F.M., Peco B. (2004). From what depth do seeds emerge? A soil seed bank experiment with Mediterranean grassland species. Seed Sci.Res..

[65] Allen R.G., Pereira L.S., Raes D., Smith M. (1998). Crop Evapotranspiration-Guidelines for Computing Crop Water Requirements.

[66] Xiao X., Horton R., Sauer T.J., Heitman J.L., Ren T. (2011). Cumulative soil water evaporation as a function of depth and time. Vadose Zone J..

[67] Karssen C.M., Khan A.A. (1982). Seasonal patterns of dormancy in weed seeds. The Physiology and Biochemistry of Seed Development, Dormancy and Germination.

[68] Hegarty T.W. (1978). The physiology of seed hydration and dehydration, relation between water stress and the control of germination: A review. Plant Cell Environ..

[69] Hudson A.R., Ayre D.J., Ooi M.K.J. (2015). Physical dormancy in a changing climate. Seed Sci. Res..

[70] Luna B., Piñas-Bonilla P., Zavala G., Pérez B. (2023). Timing of fire during summer determines seed germination in Mediterranean Cistaceae. Fire Ecol..

[71] Luna B., Pérez B., Torres I., Moreno J.M. (2012). Effects of incubation temperature on seed germination of Mediterranean plants with different geographical distribution ranges. Folia Geobot..

[72] Michel B.E., Kaufmann M.R. (1973). Osmotic potential of polyethylene-glycol 6000. Plant Physiol..

