# Supplementary material for: Hydration-Dehydration Effects on Germination Tolerance to Water Stress of Eight Cistus Species
Source: Plants (Basel). 2025 Jul 19;14(14):2237. doi: 10.3390/plants14142237 (PMC12298707; doi:10.3390/plants14142237)
Supplement: Supplementary file 1 [file plants-14-02237-s001.zip › plants-3728375-supplementary.pdf]

## Supplementary Materials

**Table S1**. Viability means (± standard error) for *Cistus* species at the different priming (control and hydration-dehydration cycles of 24 and 48 hours) and water stress treatments (0, -0.2, -0.4, -0.6 and -0.8MPa).

| Species | Water potential | Priming treatments | | |
|---|---|---|---|---|
| | | Control | 24h | 48h |
| *Cistus albidus* | 0 | 74.4 ± 4.0 | 77.9 ± 2.4 | 78.3 ± 8.5 |
| | -0.2 | 73.8 ± 3.3 | 77.5 ± 7.8 | 75.5 ± 4.7 |
| | -0.4 | 56.5 ± 5.9 | 78.3 ± 8.1 | 70.3 ± 7.1 |
| | -0.6 | 54.8 ± 8.9 | 73.6 ± 9.2 | 63.7 ± 5.2 |
| | -0.8 | 65.1 ± 3.1 | 70.5 ± 10.6 | 62.8 ± 4.9 |
| *C. clusii* | 0 | 77.1 ± 7.6 | 77.5 ± 10.6 | 79.7 ± 8.8 |
| | -0.2 | 74.8 ± 13.3 | 64.3 ± 8.3 | 76.3 ± 2.9 |
| | -0.4 | 88.7 ± 4.9 | 68.8 ± 8.1 | 71.6 ± 10.2 |
| | -0.6 | 95.7 ± 0.2 | 80.2 ± 7.0 | 68.1 ± 11.5 |
| | -0.8 | 72.1 ± 8.8 | 80.6 ± 5.4 | 82.1 ± 5.0 |
| *C. ladanifer* | 0 | 94.1 ± 1.0 | 99.1 ± 0.9 | 100.0 ± 0.0 |
| | -0.2 | 88.0 ± 3.2 | 93.0 ± 2.4 | 96.2 ± 2.2 |
| | -0.4 | 99.0 ± 1.0 | 82.5 ± 2.2 | 96.5 ± 1.2 |
| | -0.6 | 94.7 ± 2.6 | 92.7 ± 2.6 | 94.1 ± 4.8 |
| | -0.8 | 94.4 ± 4.4 | 97.8 ± 1.3 | 97.0 ± 1.9 |
| *C. laurifolius* | 0 | 74.8 ± 3.4 | 91.6 ± 4.4 | 73.1 ± 5.0 |
| | -0.2 | 66.2 ± 11.0 | 88.2 ± 2.8 | 87.0 ± 5.3 |
| | -0.4 | 76.3 ± 8.9 | 89.5 ± 5.2 | 79.0 ± 8.5 |
| | -0.6 | 45.8 ± 9.7 | 79.6 ± 4.1 | 76.1 ± 5.8 |
| | -0.8 | 44.7 ± 15.8 | 58.8 ± 12.7 | 78.7 ± 7.1 |
| *C. monspeliensis* | 0 | 97.1 ± 1.8 | 94.7 ± 2.3 | 90.6 ± 5.6 |
| | -0.2 | 97.9 ± 2.1 | 89.4 ± 2.2 | 87.3 ± 3.6 |
| | -0.4 | 54.8 ± 6.3 | 83.0 ± 5.0 | 82.5 ± 6.1 |
| | -0.6 | 78.1 ± 12.1 | 81.8 ± 8.9 | 42.8 ± 8.6 |
| | -0.8 | 82.4 ± 5.9 | 56.6 ± 16.5 | 59.4 ± 9.0 |
| *C. populifolius* | 0 | 78.8 ± 6.8 | 70.4 ± 12.8 | 78.9 ± 6.9 |
| | -0.2 | 28.7 ± 4.7 | 61.6 ± 6.3 | 83.8 ± 6.9 |
| | -0.4 | 35.0 ± 5.6 | 48.9 ± 9.1 | 63.7 ± 11.2 |
| | -0.6 | 40.3 ± 15.8 | 68.8 ± 17.8 | 32.8 ± 2.0 |
| | -0.8 | 84.0 ± 2.9 | 48.6 ± 8.5 | 21.7 ± 1.2 |
| *C. psilosepalus* | 0 | 69.7 ± 3.7 | 81.6 ± 3.0 | 67.8 ± 5.8 |
| | -0.2 | 86.6 ± 7.7 | 82.6 ± 6.0 | 57.9 ± 12.6 |
| | -0.4 | 67.4 ± 6.2 | 81.9 ± 6.1 | 70.8 ± 9.9 |
| | -0.6 | 63.3 ± 4.0 | 67.9 ± 4.8 | 82.5 ± 2.2 |
| | -0.8 | 90.7 ± 4.7 | 81.7 ± 9.6 | 81.9 ± 8.9 |
| *C. salviifolius* | 0 | 88.3 ± 2.7 | 85.7 ± 2.9 | 91.8 ± 3.0 |
| | -0.2 | 85.9 ± 5.8 | 90.7 ± 2.0 | 84.6 ± 5.1 |
| | -0.4 | 90.9 ± 3.5 | 88.0 ± 2.9 | 93.4 ± 2.7 |
| | -0.6 | 93.1 ± 1.7 | 94.9 ± 1.1 | 87.9 ± 4.9 |
| | -0.8 | 80.7 ± 2.8 | 73.9 ± 3.8 | 92.6 ± 2.2 |

**Table S2.** Results from GLM (based on a binomial error distribution and logit link function) for main effects of water stress and priming factors and their interactions on seed viability of eight *Cistus* species. Statistically significant effects ($p \leq 0.05$) are highlighted in bold, while marginally significant effects are in italics ($0.05 < p < 0.1$).

| | Water stress | | Priming | | Water stress x Priming | |
|---|---|---|---|---|---|---|
| | $\chi 2$ | *P* | $\chi 2$ | *P* | $\chi 2$ | *P* |
| *Cistus albidus* | 7.997 | *0.092* | 4.501 | 0.105 | 3.268 | 0.916 |
| *C. clusii* | 3.643 | 0.456 | 3.137 | 0.208 | 10.836 | 0.211 |
| *C. ladanifer* | 144.546 | **<0.010** | 173.011 | **<0.010** | 535.444 | **<0.010** |
| *C. laurifolius* | 16.395 | **0.003** | 16.397 | **<0.010** | 12.101 | 0.147 |
| *C. monspeliensis* | 27.716 | **<0.010** | 5.697 | *0.058* | 25.851 | **0.001** |
| *C. populifolius* | 21.037 | **<0.010** | 0.654 | 0.721 | 35.215 | **<0.010** |
| *C. psilosepalus* | 5.036 | 0.284 | 0.958 | 0.619 | 10.996 | 0.202 |
| *C. salviifolius* | 8.789 | *0.067* | 1.148 | 0.563 | 12.727 | 0.122 |

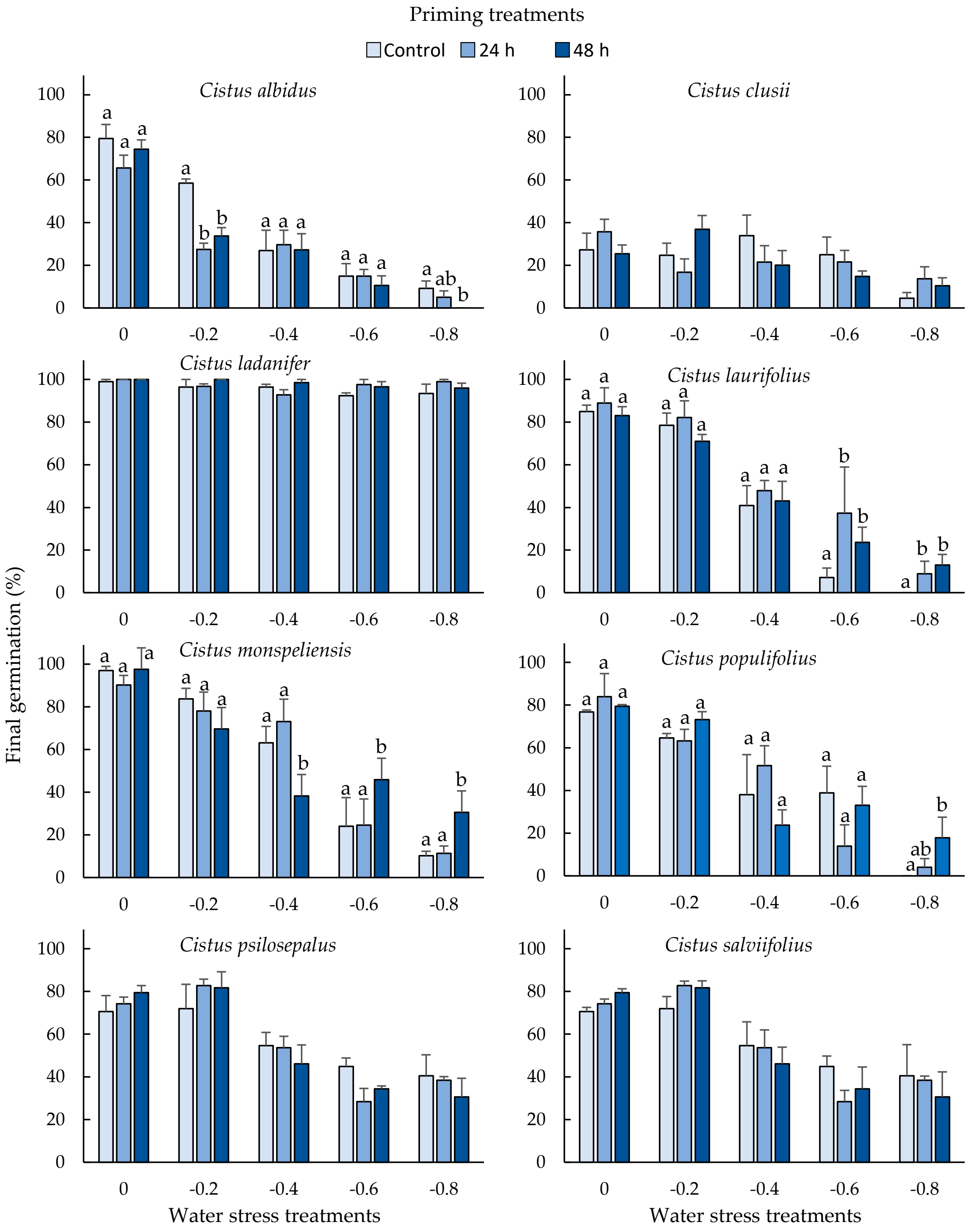


**Figure S1.** Final germination percentages (mean ± standard error) of eight *Cistus* species at the different water stress (0, -0.2, -0.4, -0.6 and -0.8MPa) and priming treatments (control and hydration-dehydration cycles of 24 and 48 hours). Different letters show significant differences ($p$ < 0.05) between priming treatments based on pairwise comparisons of Fisher's least significance difference after GLM analysis, when significant interaction between both factors emerged.
